# Brainstem and Spinal Arachnoiditis Ossificans Associated With Neurocysticercosis: A Case Report

**DOI:** 10.7759/cureus.46157

**Published:** 2023-09-28

**Authors:** Michel G Mondragon-Soto, Jaime Jesus Martinez Anda, Jose Angel Romero Figueroa, Jose Alfredo Gonzalez Soto, Roberto A De Leo Vargas

**Affiliations:** 1 Department of Neurosurgery, National Institute of Neurology and Neurosurgery “Dr. Manuel Velasco Suárez”, Mexico City, MEX; 2 Department of Neurosurgery, American British Cowdray Neurological Center, Mexico City, MEX; 3 Department of Radiology, American British Cowdray Neurological Center, Mexico City, MEX; 4 School of Medicine, Metropolitan Autonomous University, Mexico City, MEX

**Keywords:** brainstem, spine, ossification, neurocysticercosis, arachnoiditis ossificans

## Abstract

Arachnoiditis ossificans (AO), a very rare entity that can cause severe neurological deficit, is associated with an inflammatory response and compressive effect on the spinal cord.

A 65-year-old woman with diagnosis of arachnoiditis ossificans, who had a past medical history of neurocysticercosis diagnosed eight years before the actual onset that was accompanied by obstructive hydrocephalus and required bilateral ventriculoperitoneal shunts, presented with lower limb paresis. The spinal CT reported large calcified subdural spinal plaques. She was treated with high-dose steroids with subsequent improvement of the clinical manifestations.

Spinal neurocysticercosis is a rare manifestation of this disease, although the Mexican population is especially prone to it, due to the endemic presence of this entity, it can provoke spinal arachnoiditis. We conclude that the chronic inflammation of the spinal meninges induced by the cysticercosis could encourage the arachnoid cells to go through osteoblastic metaplasia with consequent production of thick calcium deposits, such as those found in AO. Thus it may be associated with AO. We present, to our knowledge, the first patient with AO-associated neurocysticercosis.

## Introduction

Arachnoiditis ossificans (AO) is a very rare condition in which the subdural arachnoid space has calcified plaques associated with arachnoiditis; in the spinal canal, it causes direct compression of the spinal cord which can originate several neurological symptoms. It differs from chronic spinal arachnoiditis by presenting with chronic proliferative changes in the meninges associated with replacement of large spinal arachnoid membranes by dense calcifications similar to bone, and an ossified shell that encases the spinal cord [1]. Very few cases of arachnoiditis ossificans have been previously described in the literature [2]. To our knowledge, our patient is the first case of AO associated with brainstem and spinal neurocysticercosis.

## Case presentation

A 65-year-old female presented to the emergency room with gait instability and nausea for 48 hours before admission. She had past medical history of neurocysticercosis eight years before being diagnosed by a non-contrast brain CT scan, a condition which resulted in hydrocephalus, and was treated with bilateral ventriculoperitoneal shunting twice several years prior to the present clinical manifestations.

**Figure 1:**
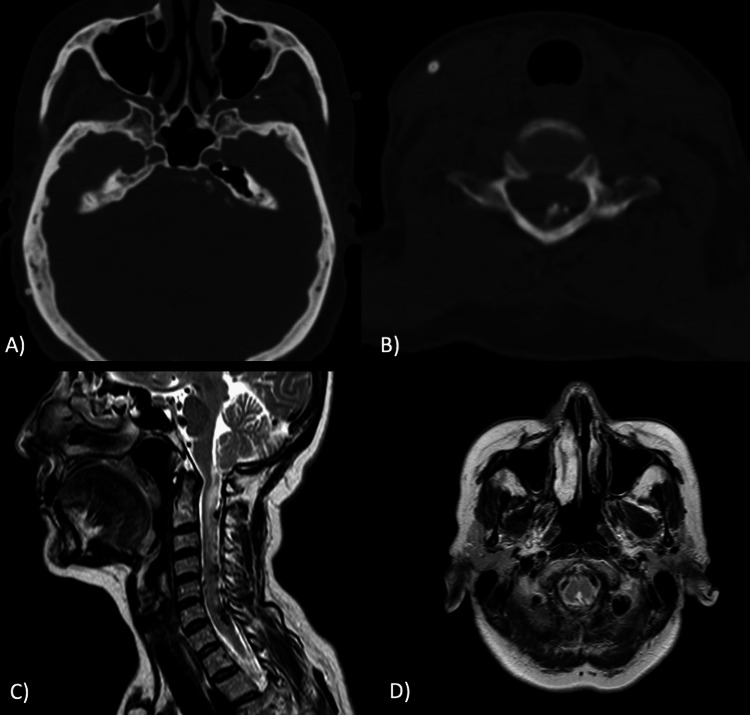
A) Axial non-contrast CT scan of the brainstem with amorphic arachnoid calcifications extending from the intracranial cisterns with cervical extension from C1 to C7 (B) with posterior predominance. C) Weighted (sagittal) and D) T2-weighted axial MRI with increased T2 hyperintense signal change within the cord with compressive myelopathy.

**Figure 2:**
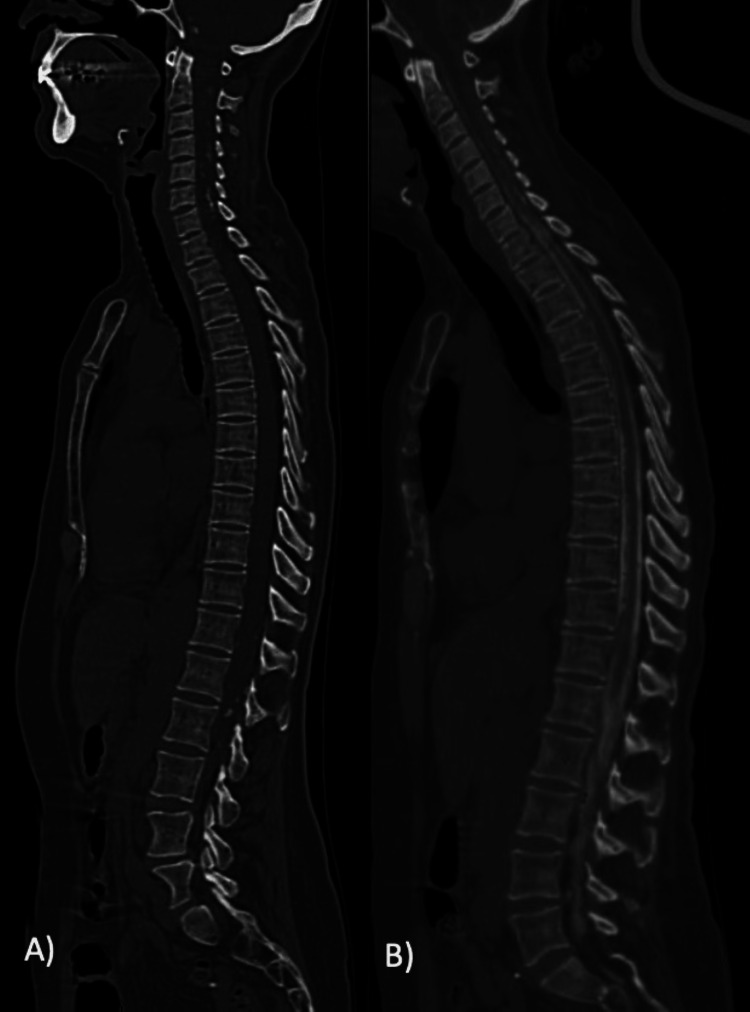
A) Non-contrast Myelotomography demonstrates amorphic arachnoid calcifications extending from the intracranial cisterns with cervical extension from C1 to C7 with posterior predominance, as well as from T11 to S1. B) Contrast Myelotomography demonstrates irregular filling of the dural sac, with a tortuous course of the nerve roots of the cauda equina. At thoracic level, there are superficial pial irregularities suggestive of an inflammatory process. At the cervical spine there are filling defects corresponding to the previously described calcifications.

On examination, she had bilateral lower extremity paresis (4/5 bilaterally), proprioceptive impairment was evident starting from the T4 dermatome, hyperreflexia and positive Babinski sign in both lower limbs; ataxic gait was also present. The Brain and whole spine computed tomography (CT) reported the existence of amorphic arachnoid calcifications at the prepontine cistern, extending to C7, and in the thoracolumbar junction from T11 to S1 level; intrathecal subarachnoid calcifications were larger from L4 to S1 levels (Figure 1). CT Myelography showed partial and irregular subarachnoid space contrast filling at the anatomical sites previously described, compatible with AO (Figure 2). The spine MRI showed no intradural lesions.

After diagnosis, high doses of steroid were initiated (prednisone 50 mg twice daily for 14 days), with improvement of neurological symptoms after the third day of therapy; treatment was continued for two weeks succeeded by titration of the aforementioned therapy was performed for the following 10 days. The steroid dose was reinitiated only when the patient reported symptoms, which happened once one year after the initial diagnosis. In a follow-up visit after 24 months, no new neurological deficit was documented. Follow-up images only included neural axis CT scan, which showed no significant changes. During this period, the patient underwent physical therapy and rehabilitation but full recovery was not achieved; the patient expressed herself as happy with the clinical results.

## Discussion

AO is a very rare pathology. Only limited case series have described its presentation, and due to the compression it exerts on the spinal cord, it can present with very florid clinical manifestations such as myelopathy and cauda equina syndrome.

Calcified leptomeningeal plaques commonly present as small calcifications, being found as incidental findings on imaging studies. Occasionally these plaques can present as thick calcifications as seen in AO [3]. It usually affects the thoracolumbar region, and rarely the cervical segment of the spine [4]. As previously stated, AO is a very rare entity that can cause severe neurological deterioration, associated with inflammatory response and compressive effect on the spinal cord; an association with syringomyelia has been reported in some cases [5,6]. These plaques can progressively compress neural elements to produce severe neurological sequelae. Patients can experience symptoms of progressive myelopathy, back and leg pain, as well as bowel and urinary incontinence, and most cases have past medical history of some kind of trauma [7].

The pathophysiology of AO is not fully understood, but it is frequently associated with chronic inflammation; after an initial insult, the inflammatory response encourages the arachnoid cells to undergo osteoblastic metaplasia with consequent production of thick calcium deposits, characteristic of AO [3]. This differs from spinal adhesive arachnoiditis in the way that proliferative changes of the meninges occur after repeated spinal anesthesia, myelography, trauma, or surgery; it is also associated with neoplasms, infectious diseases of the central nervous system and herniated intervertebral disks [1]. The only risk factors associated with our patient were the chronic infection with Taenia solium and documented neurocysticercosis, for which she received previous treatment. Taenia solium has been documented to cause spinal arachnoiditis, representing 1-2% of all cases [8], but none has been associated with AO. In the setting of chronic spinal leptomeningeal inflammation secondary to neurocysticercosis without any other inflammatory processes, the apparition of calcified plaques characteristic of AO could be explained.

Neurocysticercosis is a pathologic entity caused by the metacestod stage of the tapeworm Taenia solium, and is one of the most frequent parasitic diseases of the central nervous system (CNS), especially in endemic regions like Latin America, Asia, Central and South Africa [9,10]. Neurocysticercosis can affect different parts of the CNS, including the brain parenchyma, meninges, ventricles, eyes, and spinal cord. Spinal cord involvement is uncommon [8], but it has been shown that the Mexican population is especially prone to it and that it can provoke spinal arachnoiditis [11], thus cysticercosis can cause chronic inflammation of spinal cord meninges.

Considerable advances have been made in diagnostic imaging, reaching the current use of CT and MRI. Until 1983, before CT, the diagnosis of AO was exclusively made through surgery with the exception of one case which was done by autopsy [12]. The calcifications or ossifications in AO are typically hyperdense on CT scans and can easily be identified when compared with the hypodense thecal sac and nervous tissue [4]. On the other hand, MRI shows clumped nerve roots which is indicative of arachnoiditis. The associated calcification (or ossification) looks hyperintense on T1-weighted sequences and hypo- or hyperintense on T2-weighted images. High signals in T1-weighted and T2-weighted images may correspond to development of bone marrow [13]. Domenicucci [12] proposed a classification for the ossification of the arachnoid membrane into three types on CT scan: type I, with a semicircular or banana-like appearance most often along the dorsal aspect of the spinal cord; type II characterized by a more circular image and circumferentially surrounds the thecal sac; and type III characterized by a honeycomb pattern in which the thecal sac is traversed by the calcifications, which is crossed by the caudal roots (exclusive of the lumbar spine) [2]. In this case, our patient was classified as type II. Based upon classification, type I and II patterns are more likely to be treated with surgery, most likely a decompressive laminectomy, while type III AO is most likely treated medically. The success of medical treatment has been observed to be variable [14]. Up to date, there have not been other cases with similar risk factors reported, suggesting that the chronic inflammatory component of neurocysticercosis may predispose to the development of intrathecal plaques disseminated along the dura.

From the patient’s perspective, she reported that her symptoms improved significantly after initiating the medical treatment. Bouts of dizziness and unsteady gait are still present, which resolve rapidly after reinitiating the steroid therapy. Management of AO is controversial; whereas some advocate for aggressive surgical intervention, others argue that a more conservative management should be indicated [3,15]. Our recommendation is that every case should be individualized, and treatment should correspond to each patient’s clinical presentation. In our patient’s case, the latter was the most effective option.

## Conclusions

We present, to our knowledge, the first patient with medical history of AO associated with neurocysticercosis. Neurocysticercosis continues to be a highly prevalent disease of the CNS in endemic regions with limited sanitary services such as Mexico. It is a disease that can present with spinal cord involvement causing a chronic inflammatory response, which can consequently lead to bone deposition, resulting in AO. The choice between the different therapeutic options should be made by individualizing each case. More literature should be reported in order to confirm this association.
